# Fabrication of MIP-based sensors for femtomolar detection of kynurenic acid for early diagnosis of neurodegenerative diseases

**DOI:** 10.1021/acsomega.5c02339

**Published:** 2025-05-16

**Authors:** Kübra Turan, Gözde Aydoğdu TiĞ

**Affiliations:** Ankara University, Faculty of Science, Department of Chemistry, Ankara 06100, Türkiye

## Abstract

Neurodegenerative diseases (NDDs), among which Alzheimer’s disease (AD) is one of the most significant medical and societal challenges of this century due to its increasing prevalence, require early diagnosis through reliable biomarkers to improve disease management. Among several biomarkers, kynurenic acid (KYNA) has emerged as a newly found metabolite, identified as a sensitive and selective blood-based biomarker, with its levels increasing in the early stages of AD. In this study, we aim to develop a low detection limit, stable, low-cost, and reliable molecularly imprinted polymer-based electrochemical biosensor for the rapid, selective, and sensitive detection of KYNA, a promising biomarker for AD. For this purpose, the glassy carbon electrode was first modified with copper–silver bimetallic structures (Cu–Ag BS). Then, a 3,4-ethylene-dioxythiophene (EDOT) monomer was electropolymerized on the Cu–Ag BS/GCE in the presence of the KYNA analyte. The introduced sensor was characterized through field emission scanning electron microscopy (FESEM), atomic force microscopy (AFM), X-ray photoelectron spectroscopy (XPS), differential pulse voltammetry (DPV), electrochemical impedance spectroscopy (EIS), and cyclic voltammetry (CV). The effect of electrodeposition parameters was optimized. This is the first time the proposed MIP sensor allowed KYNA detection in a wide linear range of 1.0 fM–500 nM with a limit of detection (LOD) of 0.278 fM. Furthermore, the MIP layer provides highly selective binding by forming KYNA-specific recognition cavities. Notably, the sensor has been successfully validated in complex biological media, including fetal bovine serum and human serum, achieving high recovery values. The proposed sensor could potentially be utilized in the future design of a diagnostic kit for the early diagnosis of neurodegenerative diseases.

## Graphical abstract

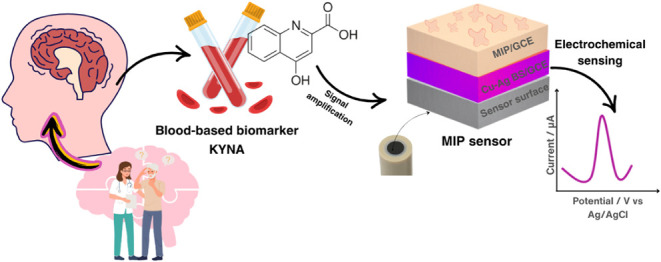

## 1 Introduction

Neurodegenerative diseases (NDDs) are a group of disorders characterized by the progressive loss of function of nerve cells in the human brain.^1^ NDDs encompass a range of diseases, including Alzheimer’s disease (AD), Huntington’s disease (HD), Parkinson’s disease (PD), amyotrophic lateral sclerosis (ALS), multiple system atrophy (MSA), and progressive supranuclear palsy (PSP).^2,3^ As the global population ages, the importance of early diagnosis and treatment of neurodegenerative diseases, and improvements in patient quality of life, is becoming increasingly evident in societies.^3^ The risk of developing NDDs and dementia increases with age. Consequently, these disorders are becoming a significant global health concern.^4–6^ The traditional methods for detecting various NDDs, including medical imaging techniques, have many disadvantages. These include low sensitivity, high cost, false positive and negative results, and the need for professionals.^5,7^ In recent years, among NDDs, the rapid increase of AD disease has attracted attention. Therefore, scientific research has focused on the discovery of new biomarkers for the diagnosis of AD, which has the highest prevalence in the population. Identifying reliable biomarkers for diagnosing AD is challenging and requires clinical evaluation of brain tissue with various imaging techniques.^4,8^ Accordingly, identifying novel biomarkers that facilitate the diagnosis and monitoring of the disease will prove invaluable in enabling the prompt commencement of treatment for patients.^7^

Another salient point for AD pertains to detectable levels of early detection biomarkers in the blood.^9^ Consequently, the optimal approach to managing the condition is to employ reliable biomarkers for early diagnosis of AD. Kynurenic acid (KYNA) has been identified as a promising candidate for the role of a sensitive and selective blood-based biomarker. The metabolism of the kynurenine pathway (KP) exerts a substantial influence on AD development and may represent a pivotal target for therapeutic interventions.^10^ One of the TRP metabolites formed from kynurenine in the KP is KYNA. It is a glial-derived metabolite, and this metabolism is activated in neurodegenerative disorders such as AD.^9,11,12^ An increase in KYNA levels has been noted in the early stages of AD.^9^ A recent report indicated that the level of KYNA in AD patients increased 1.7-fold compared to healthy individuals.^12^ The following three factors render KYNA a valuable biomarker for other NDDs, especially AD: its presence in the blood, its higher concentration in the blood compared to other AD biomarkers, and most importantly, its increased production in the early stage of the disease as it is an effective biomarker in peripheral fluids for early detection of NDD. The prevailing methodologies for accurately detecting KYNA in blood samples are based on chromatographic techniques.^9,12–14^

Various techniques have been utilized to detect AD biomarkers by traditional methods from the past to the present.^7^ Among them, electrochemical methods stand out for their superior sensitivity, ease of electrode fabrication, and considerable potential for commercialization.^15–17^ In recent years, the analysis of AD biomarkers from blood and interstitial fluid has demonstrated significant promise due to its simplicity, diagnostic accuracy (95–97%), and cost-effectiveness.^18^ In recent years, there has been a notable increase in the prevalence of AD, which has prompted significant interest within the scientific community in research aimed at facilitating early diagnosis. An accurate diagnosis is as crucial as the appropriate treatment of a disease.^19,20^ The numerous advantages of biosensor technology render it one of the most significant methods for diagnosing AD. Electrochemical biosensors are a rapid, precise, and economical option for detecting AD biomarkers. These biosensors can be coupled with diverse technologies to enhance their selectivity and sensitivity, with molecular imprinting technology being a particularly effective approach. Molecularly imprinted polymers (MIPs) can be used as a biomimetic recognition element to detect many bioactive compounds and can be integrated with different sensing methods for biosensor fabrication.^21^ MIPs are analogues of natural antibody–antigen and enzyme–substrate systems, and the ″key-lock″ mechanism is mimicked during the synthesis phase to enable the selective recognition of the target molecule.^22^ MIP is an artificial material with complementary cavities designed to bind a specific target molecule with high selectivity. MIPs offer several advantages, including high specificity, selectivity, reusability, thermal stability, and low cost, especially for biological receptors.^23^ MIPs enable easy and rapid detection of these biomarkers from different biological matrices.^21^ MIPs are superior to other common methods by offering high selectivity and low cost compared to antigens or aptamers used as recognition surfaces in conventional biosensors.^23^ Electropolymerization and chemical polymerization are the predominant methods employed in the production of MIPs.^24^ In this study, the MIP structure was formed through the electropolymerization of 3,4-ethylene dioxythiophene (EDOT), which is significantly faster and more straightforward than chemical polymerization. Furthermore, electropolymerization offers the advantage of being carried out on the surface of the working electrode, with the entire process being monitored. Additionally, the film’s thickness can be managed by varying the applied potential, making the method more reproducible and accurate,^21,23,25^ Poly-3,4-ethylene dioxythiophene (PEDOT) is one of the conducting polymers employed for MIP preparation, exhibiting high electrical conductivity, superior environmental stability, and good biocompatibility compared to other conducting polymers.^26^ The versatility of PEDOT enables its integration with nanomaterials and other polymers, thereby increasing the selectivity and sensitivity of sensing platforms. This facilitates the detection of analytes in the presence of competing species.^27^ At the same time, the study involved the deposition of two different metals in their metallic forms on the electrode surface through the bimetallic electrodeposition process. The integration of Cu–Ag bimetallic structures within the GCE modifies the resultant sensors, thereby enhancing their sensitivity, selectivity, and stability. These sensors are highly effective for a variety of applications in the field of electrochemical detection. Consequently, a cost-effective, functional, and robust electrode surface was successfully developed.

The early and reliable diagnosis of AD continues to pose a significant challenge. This is mainly due to the high cost, invasive nature, and limited accessibility of current clinical diagnostic methods. In this context, there is an urgent need in the literature to develop reliable and specific biomarkers that can be easily detected in biological fluids. In the present study, a novel, stable, and selective electrochemical sensor system has been developed for the detection of KYNA, a promising blood-based metabolite for AD diagnosis, based on a copper–silver bimetallic structure (Cu–Ag BS) and a molecular imprinted polymer (MIP). The Cu–Ag BS was electrodeposited on the GCE. Then, the PEDOT-MIP film was created using electropolymerization of EDOT. The structural and morphological properties were confirmed using many techniques. Current diagnostic strategies commonly rely on the analysis of cerebrospinal fluid (CSF), which necessitates the performance of invasive procedures such as lumbar puncture. Furthermore, advanced imaging techniques such as positron emission tomography (PET) are costly and impractical for routine clinical use. The MIP sensor developed in this study aims to overcome these significant limitations by enabling highly selective and sensitive detection of KYNA in serum, even at femtomolar levels. The system has been optimized through electrochemical techniques and offers a broad linear working range from 1 fM to 500 nM. Also, interference studies were performed to assess the selectivity of the MIP and nonmolecularly imprinted polymer (NIP) sensors. The proposed MIP-based sensor is distinguished by its high molecular recognition capability and excellent recovery rates in real sample analyses. The paucity of studies focusing on KYNA-specific, blood-based, MIP-based electrochemical sensors in the literature highlights this study’s scientific novelty and significance. In conclusion, this study addresses a critical gap in the literature by offering a cost-effective, rapid, and patient-friendly alternative for the early and noninvasive diagnosis of AD.

## 2 Experimental Section

### 2.1 Chemical and Apparatus

The redox probe was prepared using potassium hexacyanoferrate­(II) trihydrate (K_4_Fe­(CN)_6_·3H_2_O), potassium hexacyanoferrate­(III) (K_3_Fe­(CN_6_), and potassium chloride (KCl, Sigma-Aldrich). A phosphate-buffered saline (PBS) was prepared by combining sodium phosphate monobasic (NaH_2_PO_4_·2H_2_O, Sigma-Aldrich) and sodium phosphate dibasic (Na_2_HPO_4_·2H_2_O, Sigma-Aldrich). The pH of the prepared buffer was adjusted with sodium hydroxide or hydrochloric acid solutions. In the course of the interference studies, a variety of chemicals were utilized, including KYN, amyloid β (Aβ), quinolinic acid (QUIN), xanthurenic acid (XAN), Tau protein (Tau-441), l-glutamine (l-Gln), l-glutamic acid (l-Glu), l-Glutathione Reduced (GSH), and human serum albumin (BSA), all of which were procured from Sigma-Aldrich. Human serum (Sigma-Aldrich) and fetal bovine serum (Biological Industries, Haemek) were employed as real samples. All chemicals and reagents were of analytical grade and were utilized without further purification. Experimental studies were performed at room temperature. All solutions were prepared with ultrapure water. All of the other chemicals used were of analytical grade.

Electrochemical studies were conducted using an AUTOLAB AUT302N (Eco Chemie, The Netherlands) with NOVA 2.1.6 software. All electrochemical measurements were performed using a triple-electrode system. A glassy carbon electrode (BASi MF-2012) was employed as the working electrode, an Ag/AgCl electrode (BASi, MF 2079) was utilized as the reference electrode, and a platinum wire (BASi MW 1032) was used as the counter electrode. All measurements were conducted at room temperature (23 ± 2 °C). An ultrasonic bath (Bandelin, Germany) was employed to prepare dispersions and solutions. Furthermore, an AS 82/220.X2 model precision balance (RADWAG, Poland), a magnetic stirrer (HS31, CHILTERN, UK), and a pH meter (Thermo ORION 720 A, Thermo Fisher Scientific Inc., USA) were employed for the preparation of solutions and materials throughout the study. All studies obtained double-distilled water from the Purelab Option-Q DV25 system (ELGA, UK). The CV and EIS techniques were employed to obtain comprehensive data regarding the alteration of the electrode surfaces. To this end, cyclic voltammetry measurements were recorded from (−0.3 V) to (+0.6 V). DPV measurements were conducted in a potential range of (−0.3 V) to (+0.6 V), with a modulation time of 0.05 s, modulation amplitude of 0.05 V, step potential of 0.005 V, and a scan rate of 0.0336 V/s on a 5.0 mM [Fe­(CN)_6_]^3–/4–^ redox probe containing 0.1 M KCl, and EIS measurements were recorded on a sine wave with an amplitude of 5 mV in the frequency range from 0.01 Hz to 100.0 kHz. Field emission scanning electron micrographs of the electrodes were obtained using a QUANTA 400 field emission scanning electron microscope (SEM) manufactured by Philips (USA). X-ray photoelectron spectroscopy (XPS) measurements were conducted in the energy range of 100–4000 eV using a Thermo Scientific K-Alpha instrument equipped with a monochromatic Al Kα X-ray source and an 80° hemispherical analyzer-128 channel detector. The prepared electrodes’ atomic force microscopy (AFM) images were collected using the Bruker Dimension Edge with ScanAsyst.

### 2.2 Preparation of the MIP Sensor and NIP Sensor

In the initial stage of MIP sensor preparation, the surface of a bare glassy carbon electrode (BGCE) was treated with Buehler alumina (Al_2_O_3_, 0.05 μm) solution on a cleaning pad and polished with 0.1 μm, 0.25 μm, and 0.3 μm diamond polishing pastes. Subsequently, the electrode was subjected to an ultrasonic cleaning process utilizing ethyl alcohol and ultrapure water. The manufacturing stages of the proposed MIP sensor are illustrated in Scheme 1. In the initial phase of the biosensor fabrication, a highly functional and robust Cu–Ag bimetallic structure was electrodeposited on the sensor surface. The electrodeposition technique was selected as an alternative to using expensive systems to facilitate the modification process. Electrodeposited nanoparticles’ driving force and deposition rate can be readily controlled by modifying the electrode potential or current density.^28^ The electrodeposition was conducted in two distinct phases.

**Scheme 1:**
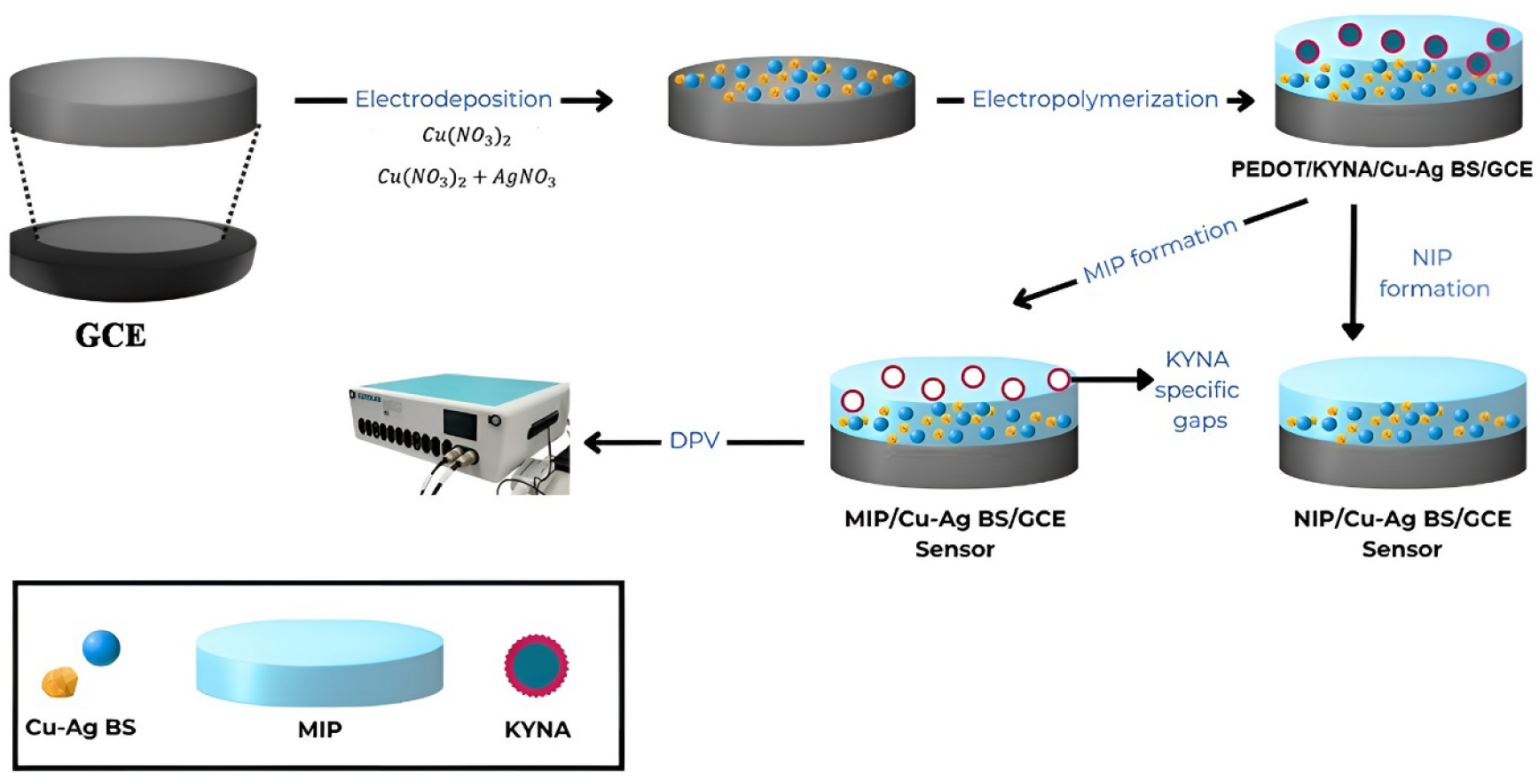
Schematic Illustration of the Proposed MIP Sensor

The Cu/GCE electrode was prepared by electrodeposition via chronoamperometry (CA) at −0.3 V for 180 s in a solution comprising 2.0 mM Cu­(NO_3_)_2_, 0.02 M thiourea, and 0.01 M H_2_SO_4_. Subsequently, cyclic voltammetry was conducted in a solution comprising 5.0 mM AgNO_3_, 2.0 mM Cu­(NO_3_)_2_, 0.02 M thiourea, and 0.01 M H_2_SO_4_,^29^ with a potential range of (−1.0 V) to (1.0 V), 10 cycles, and a scan rate of 25 mV/s.^30^ The Cu–Ag BS/GCE was washed in ultrapure water.

The subsequent stage of MIP sensor preparation involved the electropolymerization of 3,4-ethylene-dioxythiophene (EDOT) monomer on the surface of the Cu–Ag BS/GCE obtained in the prior step. To achieve this, a solution comprising KYNA (100 nM), EDOT (0.01 M), sodium dodecyl sulfate (SDS) (0.01 M), and H_2_SO_4_ (0.1 M) was electropolymerized using the CV method at a scan rate of 50 mV/s between (−1.2 V) and (+1.8 V) for a total of five cycles.^26^ SDS as a supporting electrolyte has been demonstrated to enhance the solubility of EDOT monomer in water.^31^

To complete the MIP sensor, the sensor was subjected to two distinct removal procedures. To this end, the polymer-coated electrode (PEDO*T*/KYNA/Cu–Ag BS/GCE) was maintained in phosphate buffer solution (PBS, pH 9.0) (+0.7 V) for 120 s by CA technique to remove KYNA molecules from the surface. In the second step, the formation of specific surface imprinting areas will be achieved by the CV method at a scan rate of 0.05 V/s for five cycles from (−0.5 V) to (+1.9 V).^32^ The electrode was washed with ultrapure water and designated MIP/Cu–Ag BS/GCE.

Moreover, to ascertain the efficacy of the proposed KYNA MIP sensor, a nonmolecularly imprinted polymer (NIP) sensor was fabricated using the same procedure as the MIP sensor but without including KYNA. All electrochemical measurements were carried out in a 0.1 M KCl solution containing 5.0 mM [Fe­(CN)_6_]^3–/4–^. The conditions for the CV experiment were as follows: E initial: −0.3 V, E cycle: 0.6 V, E end: −0.3 V, E step: 0.00244 V, Scan rate: 0.05 V/s. DPV conditions were as follows: E start: −0.2 V, E end: 0.6 V, E step: 0.003 V, Pulse amplitude: 0.05 V, Pulse duration: 0.02 s, Interval duration: 0.2 s, Scan rate: 15 mV/s. EIS measurements were in the frequency between 10^5^ and 0.1 Hz, Amplitude: 5 mV, Frequency number: 50 was used.

### 2.3 Real Monitoring of KYNA in Biological Fluids Using MIP and NIP Sensors

This study employed commercial serum samples for real sample analysis, with measurements performed by the DPV method. The analytical applicability of the proposed MIP and NIP sensors was investigated using DPV measurements under optimum conditions in fetal bovine serum samples from Biological Industries and human serum samples from Sigma-Aldrich using the standard addition method. Samples were diluted 10-fold without prior treatment. 100 nM KYNA was added to the sample medium by the standard addition method. The percentage recovery values were calculated.

## 3 Results and Discussion

### 3.1 The Surface Characterization of Sensors

#### 3.1.1 SEM Analysis

The electrode modifications were investigated using scanning electron microscopy (SEM). The resulting SEM images of the electrodes are presented in Figure 1. As illustrated in Figure 1A, the surface of the bare GCE is characterized by a general smoothness and uniformity. A thick and porous copper layer was observed after the deposition of copper on the surface of the GCE (Figure 1B). The deposition of Ag resulted in notable alterations to the morphology of the nanostructures. Following the electrochemical deposition of Cu–Ag BS on the Cu/GCE surface, the presence of Ag nanoparticles with nanosized particles and Cu nanoparticles with smaller sizes, which are not homogeneously distributed on the surface, is evident in Figure 1C. The codeposition of Cu and Ag resulted in the metals exhibiting regular growth along the electrode surface. The combination of Ag and Cu has a synergistic effect whereby van der Waals forces are increased. The density of recognition elements on the electrode affects the sensor’s sensitivity; thus, integrating nanomaterials on the electrode will increase the sensitivity.^33^ The electrodeposition of Cu–Ag BS increased surface area, which led to an enhancement in electrochemical sensitivity. In Figure 1D, the particles exhibited some agglomeration in the PEDO*T*/KYNA/Cu–Ag BS/GCE electrode. The size of the agglomerates of nanospheres exhibited a range of 20–50 nm. The observed agglomeration is attributed to the 3D form of PEDOT.^34^ However, SEM images of the sensors before and after KYNA cleaning (PEDO*T*/KYNA/Cu–Ag BS/GCE and MIP/Cu–Ag BS/GCE) revealed that the porosity was unchanged. The MIP/Cu–Ag BS/GCE exhibited many pronounced cavity structures, thereby providing a surface conducive to the redox reactions and diffusion of [Fe­(CN)_6_]3−/4−^35^ (Figure 1E). It is established that MIPs synthesized with nanomaterials can exhibit up to 15 times more imprinted voids than those prepared on unmodified surfaces.^33^ The presence of these cavities indicates the successful removal of KYNA from the structure of the MIP/Cu–Ag BS/GCE electrode. These findings demonstrate that the MIP electrode was successfully prepared.

**Figure 1:**
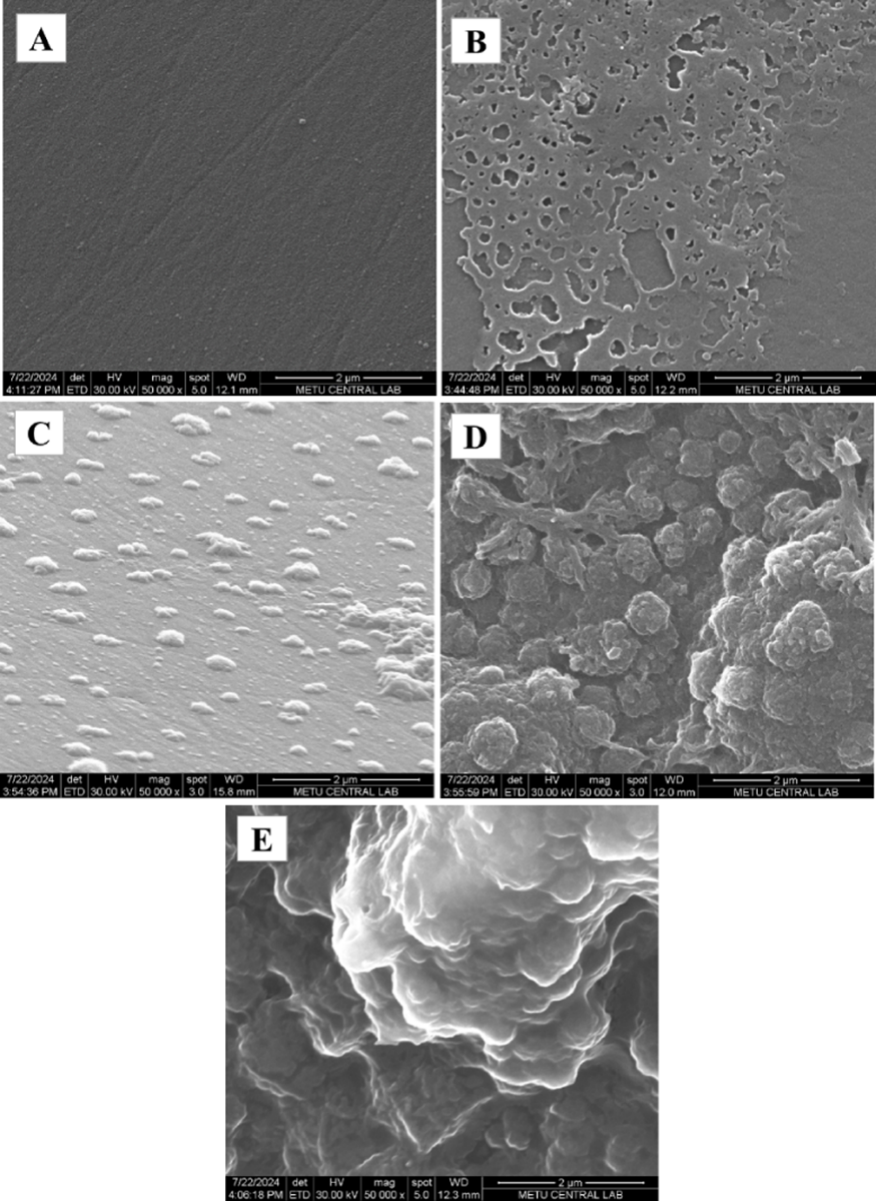
FE-SEM images of (A) Bare GCE, (B) Cu/GCE, (C) Cu–Ag BS/GCE, (D) PEDO*T*/KYNA/Cu–Ag BS/GCE, and (E) MIP/Cu–Ag BS/GCE.

#### 3.1.2 AFM Analysis

The surface topography of MIP and NIP electrodes was investigated by atomic force microscopy (AFM), with three-dimensional images recorded at room temperature on a 1 × 1 μm^2^ area. Three-dimensional (3D and 2D) topographic images of the MIP and NIP sensor surfaces are presented in Figure 2. The overall root-mean-square (RMS) roughness values were 26.6 nm for the MIP and 17.1 nm for the NIP. Compared to MIP films, the surface of the NIP film was relatively smoother.^36,37^ It was observed that the MIP surface exhibited a greater number of prominent grooves and peaks than the NIP surface. The grooves serve to confirm the presence of specific KYNA cavities. The elevated roughness of the MIP sensor indicates the existence of active sites, which facilitate electron transfer in electrochemical measurements.^38^

**Figure 2:**
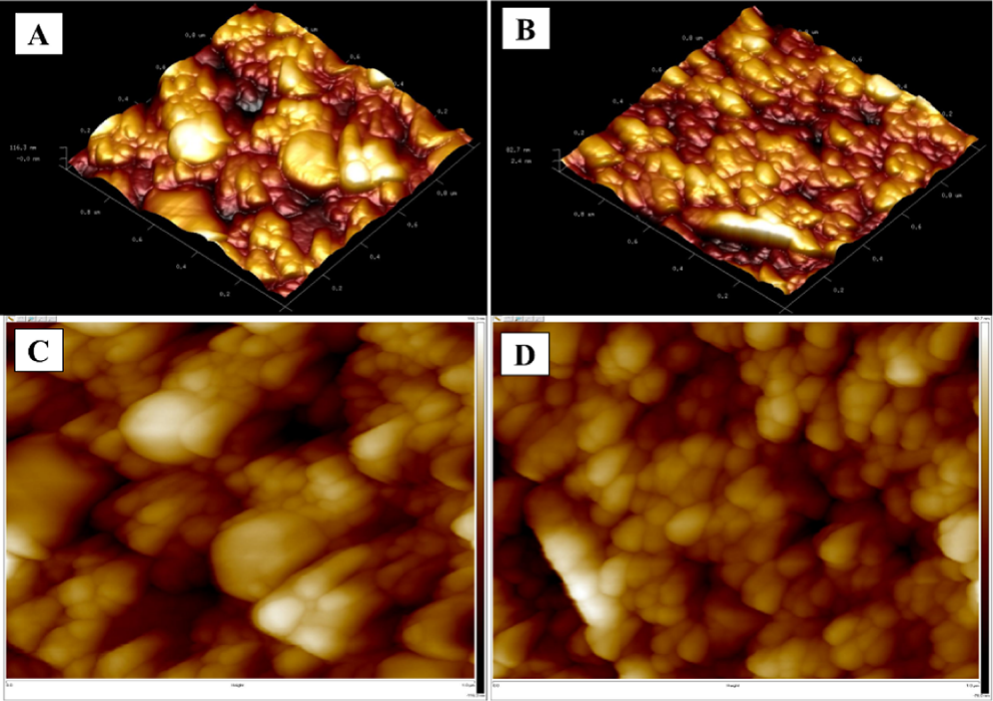
AFM images for (A) MIP sensor (3D), (B) NIP sensor (3D), (C) MIP sensor (2D) and (D) NIP sensor (2D).

#### 3.1.3 XPS Analysis

X-ray photoelectron spectroscopy (XPS) is a surface analysis technique that plays an important role in characterizing sensors and biosensors.^39,40^ The data obtained from the XPS analysis of the surface of the developed MIP sensor are presented in Figure 3. The C 1s, S 1s, O 1s, Cu 2p, and Ag 3d elements are observed in the XPS survey spectrum. Upon examination of the XPS spectrum of the prepared electrode (Figure 3A), it was observed that peaks belonging to O 1s, C 1s, Cu 2p, Ag 3d, and S 2p were present following the Cu–Ag BS deposition and PEDOT polymerization. In light of these results, it can be concluded that the modification with Cu–Ag BS and PEDOT was successfully performed. In the structure, the signals originating from PEDOT, namely C, S, and O, are also evident, as are those from Cu and Ag. As illustrated in Figure 3B, the O 1s peak exhibited three distinct peaks, with a primary peak observed at 532.9 eV, attributed to oxygen atoms forming a Cu–O bond. The remaining two peaks in the O 1s spectrum, with binding energies of 532.1 and 533.5 eV, can be attributed to PEDOT’s C=O and/or O–C=O bonds. The C 1s spectrum comprises five components, as illustrated in Figure 3C. The peaks were observed at 284.4, 284.9, 286.5, 287.8, and 289 eV. These are attributed to CC, C–C, C–S, C–OH, and CO and can be attributed to PEDOT.^41,42^ The XPS spectrum of S 2p (Figure 3D) and C 1s exhibits potential peaks that could be attributed to C–S, C=C–O, and C–O–C bonding, among others.^43^ The S 2p core-level spectrum exhibited two distinct peaks at 163.7 and 164.8 eV, which can be readily attributed to the signals of S in PEDOT, respectively. The O 1s spectrum comprises four distinct peaks at 531.8, 532.1, 532.9, and 535.7 eV, respectively. These peaks are attributed to Cu–O (lattice oxygen), CO/OC–OH, C–OH, and a π–π*.^42^ Figure 3E illustrates the Cu 2p_3/2_ peak at 933.7 eV and the Cu 2p_1/2_ peaks at 955 eV.^44^ The two principal peaks at 933.7 and 955 eV indicate Cu^+^ cations of Cu_2_O, suggesting a high-resolution Cu 2p core level spectrum.^42,43^ This finding supports the hypothesis that Cu^+^ is the predominant copper species at the electrode.^45^ The reduction process is typically associated with the formation of Cu^+^. Nevertheless, it is also conceivable that a complete reduction to Cu^0^ may occur.^46^ It has been postulated that Cu^2+^ and Cu^+^ can form coordinate bonds with electron-rich species, such as sulfur and oxygen, which are present in PEDOT.^45^ The high-resolution XPS diagram for Ag 3d, as illustrated in Figure 3F, exhibits two principal peaks at 368.3 and 374 eV, which can be attributed to the Ag 3d_5/2_ and Ag 3d_3/2_ orbitals, respectively. The splitting of the Ag 3d pair at approximately 5.7 eV corroborates the presence of Ag in its metallic form (Ag^0^), whereas the weaker peaks at 366.9 and 372.8 eV can be attributed to Ag 3d_5/2_ and Ag 3d_3/2_ of silver ions (Ag^+^). These findings illustrate the coexistence of Ag^0^ and Ag^+^ on the surface.^47^ In summary, the presence of Ag, Cu, and S supports the successful fabrication of the MIP/Cu–Ag BS/GCE electrode. The combined results of the XPS and SEM investigations provide compelling experimental evidence that the MIP/Cu–Ag BS/GCE has been successfully prepared on the GCE.

**Figure 3:**
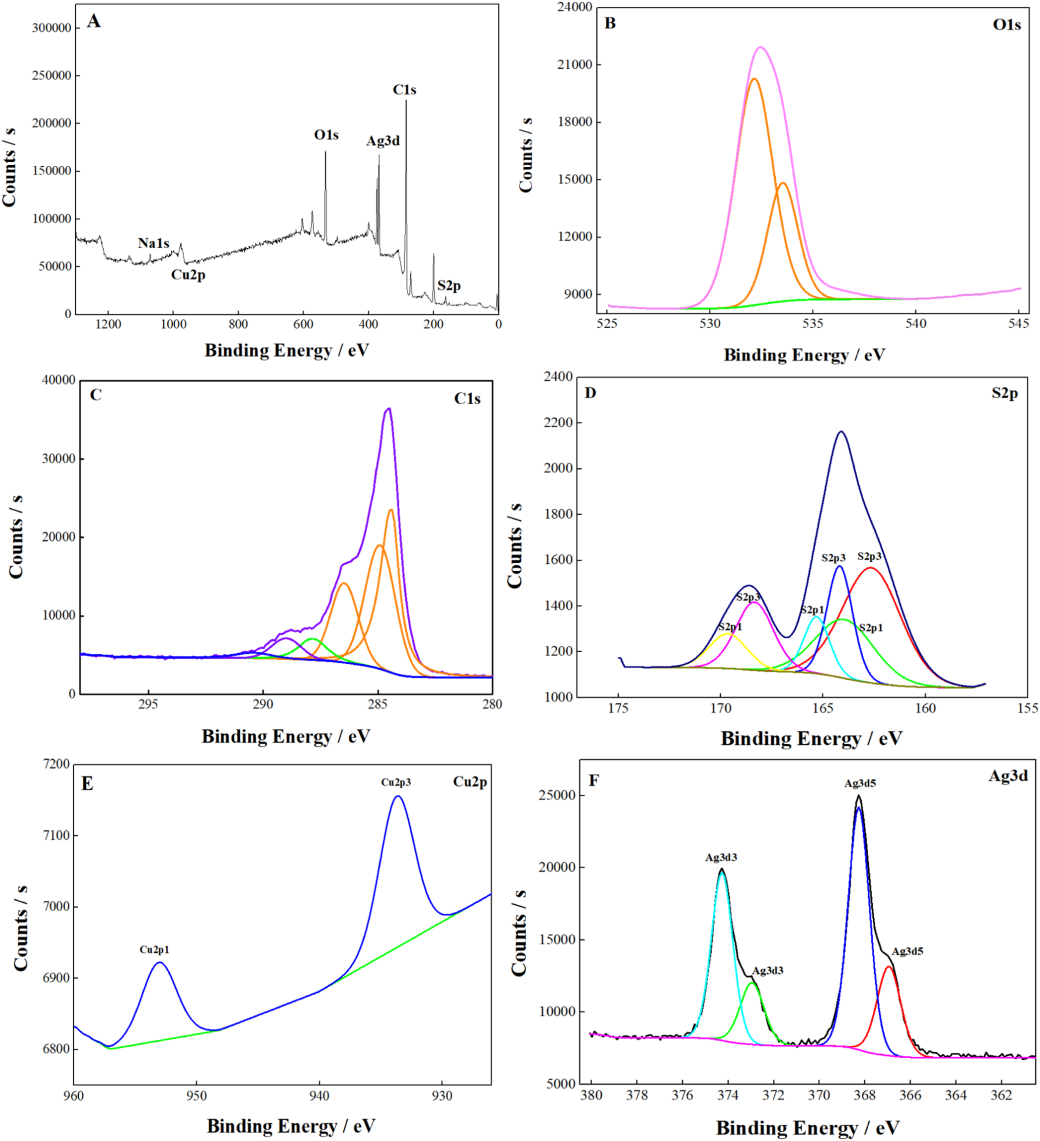
Electrode of MIP/Cu–Ag BS/GCE: (A) XPS survey spectrum, (B) XPS spectrum of O 1s, (C) C 1s, (D) S 2p, (E) Ag 3d, and (F) Cu 2p.

### 3.2 The Electrochemical Characterization of Sensors

#### 3.2.1 CV Characterization

Electrochemical characterization of the bare GCE, Cu/GCE, Cu–Ag BS/GCE, PEDO*T*/KYNA/Cu–Ag BS/GCE, MIP/Cu–Ag BS/GCE, KYNA/MIP/Cu–Ag BS/GCE, and NIP/Cu–Ag BS/GCE electrodes was conducted using a redox probe solution. CV and EIS measurements were performed, and anodic and cathodic peak currents (*i*_pa_, *i*_pc_), peak-to-peak separation values (Δ*E*_p_), and *R*_ct_ values were calculated for each electrode. The results were then compared (Table S1). Figure 4A illustrates the alternating voltammograms of the bare and modified GCE electrodes in a 0.1 M KCl solution containing 5.0 mM Fe­(CN)_6_^3‑/4–^. Figure 4B presents a comparison of the MIP and NIP sensors. Compared to the bare GCE, the Cu, and Cu–Ag BS modifications increased the electrodes’ anodic and cathodic peak currents (Figure 4A-b,c). The presence of the Cu–Ag bimetallic system has been observed to enhance the electroactivity of the electrode. This can be attributed to Ag–Cu bimetallic nanoparticles’ excellent electrical properties and larger surface area.^48^ The Cu–Ag BS/GCE electrode exhibits a higher peak current than the Cu/GCE electrode. The enhanced current response can be attributed to the superior conductive properties and larger surface area of the Ag and Cu nanoparticles.^49^ The Cu–Ag BS/GCE electrode exhibited the highest peak current among the seven electrodes (Figure 4A-c). At a peak-to-peak separation of approximately 220 mV for the bare GCE, a sharp decline to 83 mV was observed. The peak current of the Cu–Ag BS/GCE electrode is approximately 2.1 times that of the GCE electrode. The current response of the PEDOT-KYNA/Cu–Ag BS/GCE electrode decreased significantly with the electropolymerization process (Δ*E*_p_ = ∼154 mV). This can be attributed to the reduction of conductivity due to trapping the nonconductive target template in the conducting polymer PEDOT.^50^ Following the formation of the MIP at the MIP/Cu–Ag BS/GCE electrode, KYNA was moved away from the surface, forming template cavities that would assume the shape of the KYNA metabolite. A notable increase was observed in Δ*i*_p_, which rose from approximately 21 μA to 155.5 μA. The formation of holes in the insulating layer, which was created through electropolymerization, increased electron transfer, thereby enhancing the peak current.^51^ Figure 4A-f illustrates that the peak current of the KYNA/MIP/Cu–Ag BS/GCE electrode is lower than that of the MIP/Cu–Ag BS/GCE electrode. This is due to the immobilization of KYNA on the surface, which inhibits electron transfer. It can be concluded that KYNA is immobilized on the electrode surface and readily reconnected. As illustrated in Figure 4B, a comparison of the prepared MIP and NIP sensors revealed that the MIP/Cu–Ag BS/GCE sensor exhibited a higher current value and a lower Δ*E*_p_ value. It can be concluded that the modifications have significantly enhanced the redox reaction and expanded the electroactive surface area of the electrode.

**Figure 4:**
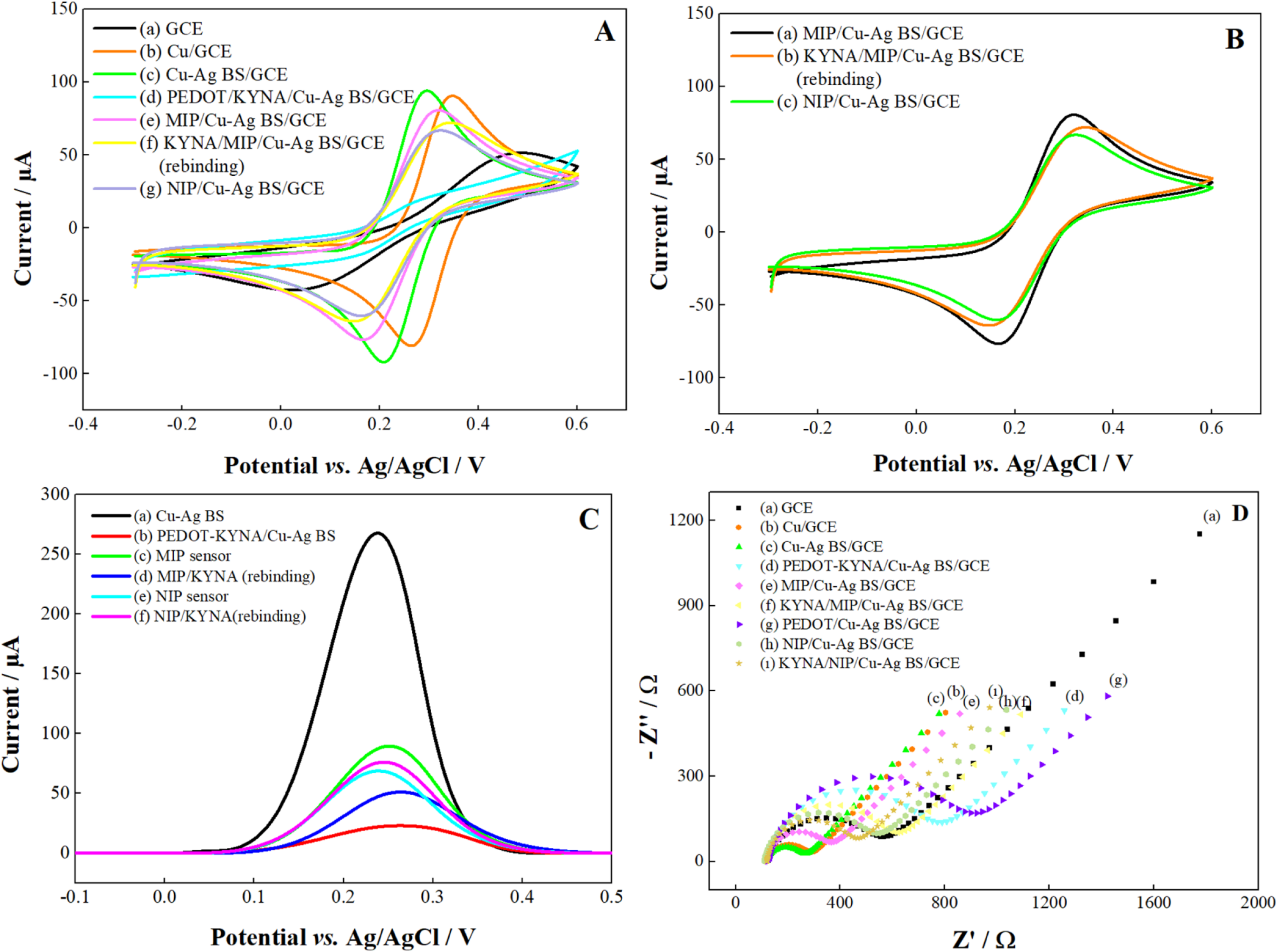
(A) CVs and (C) DPVs of different electrodes (50 mV/s scan rate, in 0.1 M KCl solution containing 5.0 mM Fe­(CN)_6_^3–/4–^). (B) CVs of (a) MIP/Cu–Ag BS/GCE, (b) KYNA/MIP/Cu–Ag BS/GCE, and (c) NIP/Cu–Ag BS/GCE electrodes. (D). Nyquist curves obtained from the different electrodes (5 mV perturbation, 10^5^ −10^–2^ kHz, in 0.1 M KCl solution containing 5.0 mM Fe­(CN)_6_^3‑/4–^) fitted with the Randles equivalent circuit model.

#### 3.2.2 DPV Characterization

Furthermore, the electrochemical behavior of KYNA was investigated on a range of modified electrodes using the DPV technique within the redox probe. As illustrated in Figure 4C, an oxidation peak exhibited a more pronounced higher current response in the MIP sensor than in the NIP sensor. The diminished peak current observed in the voltammogram obtained before the MIP synthesis, as illustrated in Figure 4C-b, can be attributed to the complete electropolymerization of the PEDOT polymer within the structure, which impedes electron transfer. The peak current of the MIP/KYNA electrode is lower than that of the MIP electrode due to the inhibition of electron transfer by KYNA immobilized on the surface. As a result of the rebinding of KYNA, rapid filling of the gaps occurs, which ultimately restricts the diffusion of redox probe ions in the area close to the electrode. This leads to a reduction in the rate of redox reactions of the probe, which is reflected in a decrease in the current (as illustrated in Figure 4C-d).^52^ It can thus be concluded that KYNA is immobilized on the electrode surface and readily reconnected. The same outcomes were observed in the CV characterization. Figure 4C-e illustrates the opposite NIP/KYNA (rebinding) electrode situation. The accessibility of the redox probe ions toward the electrode surface resulted in fewer KYNA-specific gaps being filled or absent during the rebinding event, which, in return, led to a higher current response.

#### 3.2.3 EIS Characterization

EIS characterizations of the electrodes were carried out using a redox solution containing 5.0 mM Fe­(CN)_6_^3‑/4–^ and 0.1 M KCl. The impedance measurements were taken at a frequency range of 100 kHz and 0.01 Hz with an amplitude of 5 mV and an open circuit potential. Figure 4D illustrates the Nyquist curves of the bare and modified GCE electrodes modeled using the Randles equivalent circuit model. This model includes the electron or charge transfer resistance (*R*_ct_), the ohmic resistance (*R*_s_) of the electrolyte solution, the double layer capacitance (C_dl_), and the Warburg impedance (Z_W_) caused by the diffusion of ions from the bulk solution to the electrode surface.^53^ The diameter of the semicircle observed at high frequencies in Nyquist curves is equal to the *R*_ct_ value. Using these curves, the *R*_ct_ values for each electrode of the Randles circuit were calculated. Here, the *R*_ct_ values for the bare GCE, Cu/GCE, Cu–Ag BS/GCE, PEDO*T*/KYNA/Cu–Ag BS/GCE, MIP/Cu–Ag BS/GCE, KYNA/MIP/Cu–Ag BS/GCE, PEDOT/Cu–Ag BS/GCE, NIP/Cu–Ag BS/GCE, and KYNA/NIP/NIP/Cu–Ag BS/GCE electrodes were calculated as 576.70 Ω, 164.76 Ω, 144.22 Ω, 619.56 Ω, 222.19 Ω, 460.39 Ω, 735.56 Ω, 399.86 Ω, and 315.41 Ω, respectively. Figure 4D shows that the diameter of the largest semicircle and *R*_ct_ values belong to PEDOT/CYNA/Cu–Ag and PEDOT/Cu–Ag BS/GCE electrodes. The large semicircle indicates very slow electron transfer. The electropolymerization process prevented charge transfer from the polymer-coated surface. On the contrary, it can be said that when the electrode surface is coated with nanoparticles, semicircles with smaller diameters and linear parts are obtained when the electrode surface is coated with nanoparticles (Figure 4Da–c). The lower *R*_ct_ value indicates an increase in the electron transfer rate at the solution/electrode interface. This can be interpreted as nanoparticles increasing the electrode conductivity. According to the results, it is seen that the modifications made cause changes on the surface of the electrodes at each step. As can be seen from Figure 4D-e, h, according to the *R*_ct_ values obtained for the MIP/Cu–Ag BS/GCE (*R*_ct_ = 222.19 Ω) and NIP/Cu–Ag BS/GCE (*R*_ct_ = 399.86 Ω) electrodes, it can be clearly stated that the MIP procedure enhanced the electrode conductivity and optimized the electrochemical properties. The *R*_ct_ value (460.39 Ω) obtained when KYNA rebinding was performed was higher than that of the MIP electrode, proving that the template molecules were successfully immobilized in the specific cavities. This is also supported by the fact that the same situation was not observed for the NIP sensor. The results obtained from EIS, DPV, and CV were parallel. These results confirmed the successful preparation of MIP/Cu–Ag BS/GCE and its potential for use in electrochemical sensing applications.

#### 3.2.4 The Scan Rate and Electrochemical Surface Area

In electrochemistry, both the scan rate and the electrochemical surface area are crucial parameters affecting the behavior and performance of electrochemical systems. The effect of the scan rate on the electrocatalytic properties of the redox couple was investigated using the CV technique. When the scan rate is varied, the diffusion layer thickness can change, which affects the current response. This distinguishes between a diffusion-controlled reaction and one that is kinetically controlled. For this purpose, voltammograms were recorded for the prepared electrodes against an Ag/AgCl electrode in a redox solution within a potential range of (−0.2 V) – (+0.6 V) at scan rates of 10–250 mV/s. The resulting graphs are presented in Figure S1. When the anodic and cathodic peak currents were plotted separately against the square root of the scan rate using the data in the figure, it was observed that the curves obtained were linear. This demonstrates that the electron transfer process at the solution–electrode interface is diffusion-controlled.^54,55^ Electroactive surface area (EASA) describes the effective surface area of an electrode that is available for electrochemical reactions. A larger EASA indicates more active reaction sites and a higher catalytic effect. The electroactive surface area of the sensor was determined using the Randles-Sevcik eq 1 below.^31,56–58^ The EASA values obtained for all sensors are presented in Table S2. While the EASA of Cu–Ag BS/GCE was calculated to be 0.113 cm^2^, the surface area of the bare GCE was calculated to be 0.071 cm^2^. This modification with nanoparticles resulted in a 1.6-fold increase in the surface area of the electrode. Moreover, as predicted, it caused a significant decrease in surface area due to blocking the active sites after electropolymerization. In the KYNA/MIP/Cu–Ag BS/GCE electrode, after the removal of KYNA, the cavities opened in the insulating layer, increasing the surface area. On the other hand, the MIP/Cu–Ag BS/GCE electrode exhibited a lower surface area than that of the MIP electrode because KYNA successfully reattached to the electrode surface. This is evidence of its successful immobilization onto specific cavities in the template. The surface area of 0.104 cm^2^ obtained for the MIP sensor is larger than that of the surface area of 0.092 cm^2^ of the NIP sensor. These values indicate that the MIP procedure enhances the electrode conductivity and improves the electrochemical properties.

1 $$i_{p}=\left(2.69\times 10^{5}\right)n^{3/2}ACD^{1/2}v^{1/2}$$

In this equation, the variables are defined as follows: *i*_p_ is the peak current (A), n is the number of reacting electrons (*n* = 1), A is the electrode surface area (cm^2^), C is the [Fe­(CN)_6_]^3–/4–^ concentration in solution (mol cm^–3^), D is the diffusion coefficient, and υ is the scan rate (V s^–1^) (υ = 0.05).

### 3.3 Optimization of the Experimental Conditions for the MIP Sensor

#### 3.3.1 Copper Electrodeposition Optimization

The Cu–Ag BS/GCE electrode was prepared in two distinct stages. In the initial phase, copper electrodeposition was conducted on the surface utilizing the CA technique, forming the Cu/GCE electrode. It is essential to optimize two key parameters to enhance the electrodeposition process. This step has a significant impact on the analytical performance of the electrode. To this end, the working electrode was applied with a potential of −0.3 V against Ag/AgCI for 180 s in a solution comprising 0.02 M thiourea and 0.01 M H_2_SO_4_ with differing copper concentrations. The redox response of each electrode prepared at varying concentrations was evaluated. Figure S2A illustrates the voltammograms of Cu/GCE electrodes prepared at different concentrations, plotted as a function of copper concentration versus current. In general, an increase in copper concentration resulted in a reduction in the current response. Accordingly, 2 mM Cu­(NO_3_)_2_ was identified as the optimal copper concentration (Figure S2A-c). The electrodeposition time is as crucial as the concentration in this study. As the electrodeposition time increases, the electrode surface’s layer thickness also increases. To determine the optimum time, the effect of each electrode prepared at different times on the redox response was evaluated (Figure S2B). The optimum electrodeposition time was determined to be 180 s, with the best response. In the following section, Cu/GCE electrodes were prepared with 2 mM Cu­(NO_3_)_2_, the optimum copper solution, at 180 s.

#### 3.3.2 Copper–Silver Bimetallic Structure Electrodeposition Optimization

Electrodeposition of the bimetallic nanostructures is the process of depositing two dissimilar metals on an electrode, and the metal ions are deposited on the electrode surface in metallic form. The formation of bimetallic alloy nanoparticles comprising different metals has resulted in a significant performance enhancement compared to pure metals. Concurrently, electrodeposition is recognized as one of the most practical methods for preparing nanostructured and bimetallic materials, and it is the most widely used technique commercially.^28^ Copper–silver bimetallic nanostructures are particularly interesting due to their noteworthy catalytic and electrocatalytic properties, which exhibit superior cost-effectiveness compared to other noble metals such as gold, platinum, and palladium.^59^ The fabrication of the Cu–Ag BS/GCE electrode involved the electrodeposition of Cu–Ag on the Cu/GCE electrode at varying Ag concentrations, as detailed in the section *preparation of the MIP sensor and NIP sensor*. Figure S3A illustrates Cu–Ag BS/GCE electrode voltammograms prepared at varying silver concentrations. Upon examination of the figure, it becomes evident that redox peaks belonging to electrodes modified with bare GCE are present. A comparison of the redox peaks with those of the bare GCE revealed that the *i*_pa_ and *i*_pc_ values obtained at the modified electrodes were higher. The solution concentration of 2 mM Cu­(NO_3_)_2_:5 mM AgNO_3_ was identified as the optimum. The impact of the number of bimetallic electrodeposition cycles on the sensors is of significant consequence for the sensitivity and performance of the sensors. As the number of cycles increases, there are notable alterations in the thickness and homogeneity of the metal layers deposited during the electrodeposition process. Such alterations have a direct impact on the functionality of the sensors. Accordingly, the effect of the number of cycles on the electron transfer characteristics of the modified electrodes was examined using the CV method. The voltammograms obtained for Cu–Ag bimetallic electrodes prepared at different cycles are presented in Figure S3B. At the 10th cycle, the highest current response was observed (Figure S3B-c). The observed increase in current response can be attributed to a thicker Cu–Ag BS layer providing a larger surface area to the electrode. However, as illustrated in Figure S3Bd–f, when the electrodeposition thickness is excessive, this effect is reversed, resulting in a decline in electrocatalytic activity. The Cu–Ag BS/GCE electrode prepared with 10 cycles exhibited a higher redox Δ*E*_p_ and a superior electrocatalytic effect than the other electrodes. Accordingly, the number of cycles was identified as 10 as the optimal value. Subsequently, the electrodes were electrodeposited for 10 cycles.

#### 3.3.3 MIP Optimization

The simplicity and accessibility of controlling the MIP thickness represent a significant advantage of surface-formed MIPs. The thickness of the MIP film is significantly influenced by the number of cycles employed in the electropolymerization process.^33^ The electropolymerization of EDOT can be achieved at relatively low oxidation potentials, facilitating the direct formation of uniform PEDOT films on electrode surfaces. The incorporation of PEDOT enhances the stability and sensitivity of sensors through several mechanisms. In order to achieve this, PEDOT electropolymerization was conducted at 2, 5, 10, and 20 cycles, as outlined in the section of the *preparation of the MIP sensor and NIP sensor*. The optimal number of cycles was identified by comparing the differential values (ΔI) between the oxidation peak current (I_0_) of the redox probe obtained by removing KYNA from the surface in the DPV method and the oxidation peak current (I) of the redox probe obtained after KYNA rebinding. In summary, the optimal number of cycles was identified by monitoring the fluctuations in ΔI (ΔI = I_0_-I). The results are presented in Figure S4A. Upon analysis of the data, it became evident that the KYNA reconnection process was not executed optimally, as evidenced by the formation of thin MIP layers at the second cycle and a notable decline in sensor responses. As the number of cycles increases to 5 or higher, the ΔI value gradually declines. This may be due to the inefficient extraction process caused by the thickened polymer layer. Consequently, the optimal number of cycles for PEDOT electropolymerization was identified as five, at which the highest ΔI value was attained, and a stable polymer film layer was formed.

#### 3.3.4 Removal Optimization

A further crucial stage in the evolution of MIP-based sensors is the elimination of the template, that is to say, the target analyte, from the structure.^60^ The removal of the template represents a critical step in regulating the density of the imprinted cavities.^33^ Various techniques may be employed to remove trapped target analytes, including chemical or enzymatic methods and electrodeposition.^60^ In this study, the removal of KYNA molecules from the PEDO*T*/KYNA/Cu–Ag BS/GCE surface, as detailed in the section *preparation of the MIP sensor and NIP sensor*, in two stages. The CA^32^ and the CV techniques employed a PBS solution (pH 9.0) as the removal solution. To determine the optimal number of cycles for the CV technique, the removal solution was subjected to 2, 5, 10, and 20 cycles until the formation of the MIP was complete. Figure S4B depicts the cyclic voltammograms obtained during the removal of the KYNA molecule. The greatest change in current was observed following the application of five cycles. Subsequent cycles of treatment with the removal solution resulted in a reduction in the electrode response. Accordingly, the number of cycles was identified as five, which was the optimal number.

#### 3.3.5 Rebinding Optimization

The template rebinding process directly impacts the developed sensor’s analytical capability and sensor validation.^61^ Once the MIP/Cu–Ag BS/GCE sensor was fabricated, the optimal time required for the rebinding of KYNA to the surface was investigated. The binding of 100 nM KYNA to specific cavities was investigated at different incubation times, with a range of 10 to 30 min, as illustrated in Figure S4C. Upon analysis of the ΔI values, it was observed that specific cavities may have reached saturation after 20 min, resulting in a subsequent decline in the ΔI value. The optimal binding time of 20 min was selected as the most effective for achieving stable binding of KYNA to specific cavities on the printed surface and for reusing the developed sensor for its intended purpose.

### 3.4 Evaluation of the Analytical Performance of the MIP Sensor

To assess the analytical performance of the developed MIP sensor, DPV measurements were conducted by interacting increasing concentrations of KYNA with the MIP/Cu–Ag BS/GCE and the NIP/Cu–Ag BS/GCE. Under optimal conditions, the current resulting from the oxidation of KYNA exhibited a progressive decline with increasing KYNA concentration. These findings align with those reported in numerous studies in the literature.^33,37,52,62–64^ The decrease in current value can be attributed to the closure of the electroactive surface resulting from the coupling of KYNA with MIP cavities.^63,64^ The rebinding of KYNA to the imprinted cavities reduces the number of sites available for the redox probe to reach the electrode surface,^33,52^ thereby resulting in a reduced current response. Figure 5A depicts the DPV plot, while Figure 5B illustrates the calibration plot. Instead of using absolute current values, which can vary with electrode surface areas and electrode configurations, the calibration plot is presented as log­[KYNA] concentration versus relative current change (ΔI [ΔI = I_0_ – I]). In this context, I_0_ and I represent the current values obtained as a result of DPV measurement in the absence and presence of the specific KYNA molecule, respectively. A comparison of the MIP and NIP sensors was conducted on the calibration graph. The preparation of particular cavities for the MIP sensor has resulted in a notable enhancement in the sensor’s analytical performance. Figure 5B illustrates that the current response of the NIP sensor remains largely unaltered. The absence of specific cavities printed for KYNA in the NIP sensor resulted in the analyte not rebinding at the desired rate. The limit of detection (LOD) and limit of quantitation (LOQ) values were calculated using the following eqs 2,3, respectively:

2 LOD=3s/m

3 LOQ=10s/m

s: standard deviation of the current values obtained for the lowest concentration in the calibration curve (*n* = 3); m: slope of the calibration curve.^65–68^

**Figure 5:**
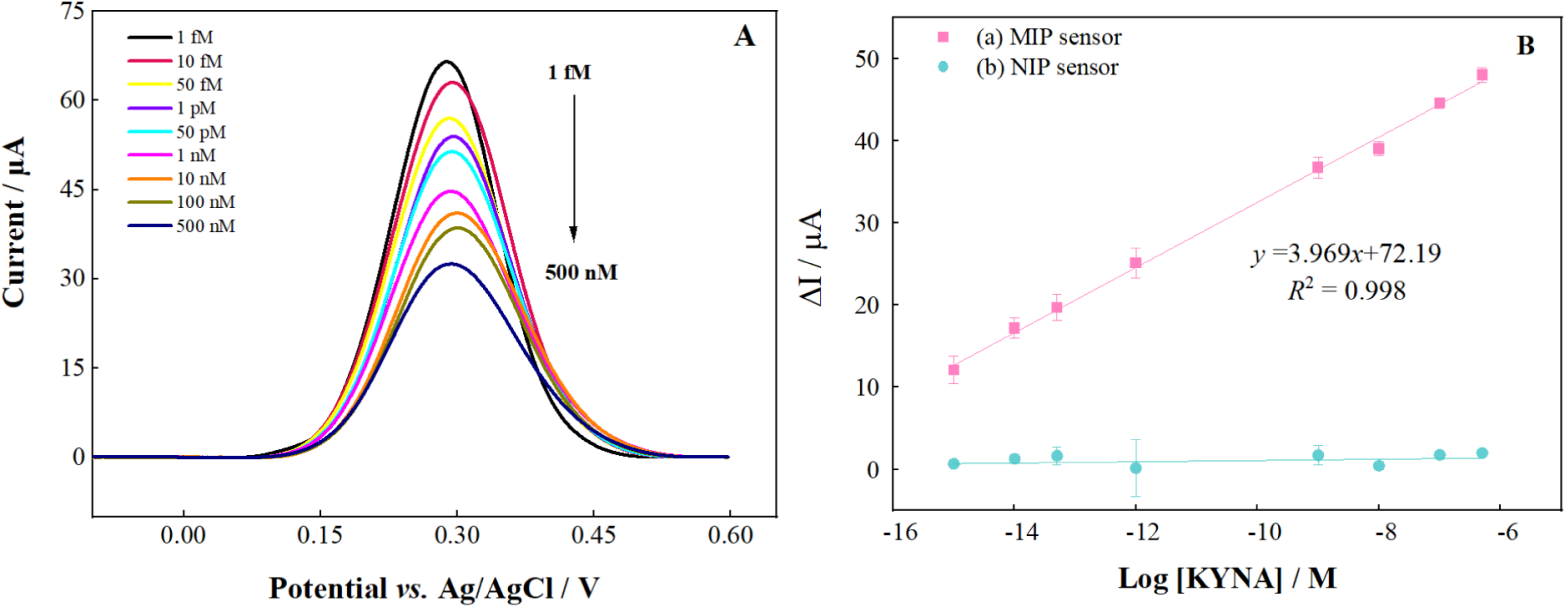
(A) The differential pulse voltammograms obtained for KYNA with the MIP sensor in the concentration range of 1 fM–500 nM and (B) calibration curves obtained for KYNA with (a) MIP and (b) NIP sensors in the concentration range of 1 fM–500 nM.

The calibration plot reveals that the oxidation of the analyte has a good linear relationship with its concentration (*R* ^2^ = 0.998). The LOD was 0.278 fM, while the LOQ was found to be 0.928 fM, which is a relatively low concentration. The linear working range of 1 fM to 0.5 μM is notably expansive. The LOD, LOQ values, and linear operating range calculated in this project are compared with those obtained from the literature studies in Table S3. When the table is analyzed, it is concluded that the MIP/Cu–Ag BS/GCE electrode developed in the project can successfully determine KYNA with a very low detection and determination limit and a wide operating range. In addition, the developed sensor has superior properties compared to the referenced studies in the literature.

Recently, biosensors have been developed to detect neurological diseases (Table S3). A detailed examination of the table reveals that sensors with diverse modified systems are predominantly developed against the biomarker Aβ. In all three studies,^5,69,70^ the limit of observability is at fM levels. Marrugo-Ramírez et al. reported an LOD of 278.8 pM for the target analyte KYNA in human serum. This finding indicates that detecting KYNA in blood samples is feasible.^9^ In a recent study by Bornaei et al., a sensor for KYNA was developed. The LOD is at the nM level in the study. The most significant disadvantage associated with utilizing the proposed sensor for quantifying KYNA is that the operational pH range is constrained to 1.0. The utilization of this pH in biological fluids is likely to impede practical measurements, as it may result in the denaturation and precipitation of proteins in an acidic environment.^71^ To the best of the authors’ knowledge, no MIP-based biosensor platform exists in the literature for the sensitive and selective determination of KYNA. The KYNA MIP biosensor developed in this study was produced at a lower cost and with higher selectivity than the immunosensor,^9^ achieved without antibodies. Furthermore, the MIP-based KYNA biosensor is comparatively more straightforward to fabricate. In this study, the accuracy of the MIP sensor was tested with the NIP sensor. The conclusion drawn from this study is that the newly developed MIP-based biosensor represents a significant contribution to the existing body of knowledge in this field.

### 3.5 Interference Study

The effect of interfering species on MIP and NIP sensor response was analyzed separately. The selectivity of the sensors prepared in the study was investigated in media containing other biomarkers, including KYN, amyloid β (Aβ), quinolinic acid (QUIN), xanthurenic acid (XAN), Tau protein (Tau-441), l-glutamine (l-Gln), l-glutamic acid (l-Glu), l-Glutathione Reduced (GSH), and human serum albumin (BSA). These molecules have been frequently identified with KYNA.^5,71–82^ The interfering species and KYNA molecule were added simultaneously at a concentration of 100 nM, and the DPV measurements were performed after rebinding to the sensor surface for 20 min. The sensor response for KYNA was also analyzed and plotted according to the percentage of relative current. As illustrated in Figure 6A, the KYNA signal in the MIP sensor exhibited no discernible interference effect. A current response within 97.19% to 102.62% was observed in the presence of interfering species. Of the species examined, the highest interference rate was observed for Aβ, with a value of 2.61%. The values in this range are below the accepted limit of 5%, as recommended in the literature.^71^ The results demonstrated that the MIP sensor exhibited high selectivity for determining KYNA. The results for the NIP sensor are presented in Figure 6B. It was observed that, except for BSA, XAN, and Tau-441, the other species exhibited a considerable impact on DPV responses. This indicated that the electrode surface produced through molecular imprinting markedly enhanced its selectivity for the target analyte, KYNA.

**Figure 6:**
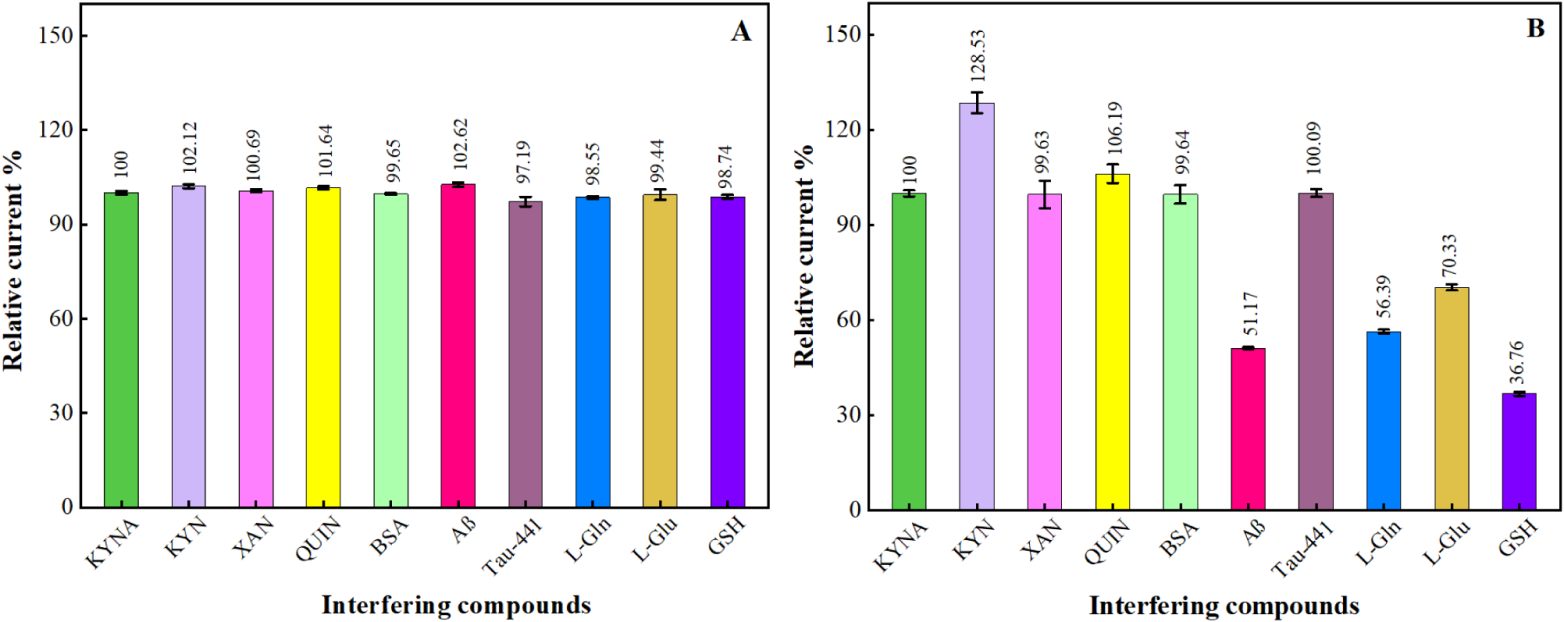
Selectivity of the proposed (A) MIP and (B) NIP sensors against KYNA in the presence of various interfering species.

### 3.6 Repeatability, Reproducibility, and Storage Stability of the MIP Sensor

MIP and NIP sensors were prepared to investigate the repeatability under optimum conditions for determining KYNA. The DPV measurements of the prepared MIP and NIP sensors were conducted five times consecutively, with a 2 min interval between each measurement. The results are presented as a bar graph in Figure 7A. The relative standard deviation % (RSD%) value was calculated from the peak currents of the voltammograms obtained. The RSD% for the MIP sensor was 1.05%, while that for the NIP sensor was 1.24%. Therefore, it can be concluded that the responses of the prepared electrochemical sensors demonstrate high repeatability.

**Figure 7:**
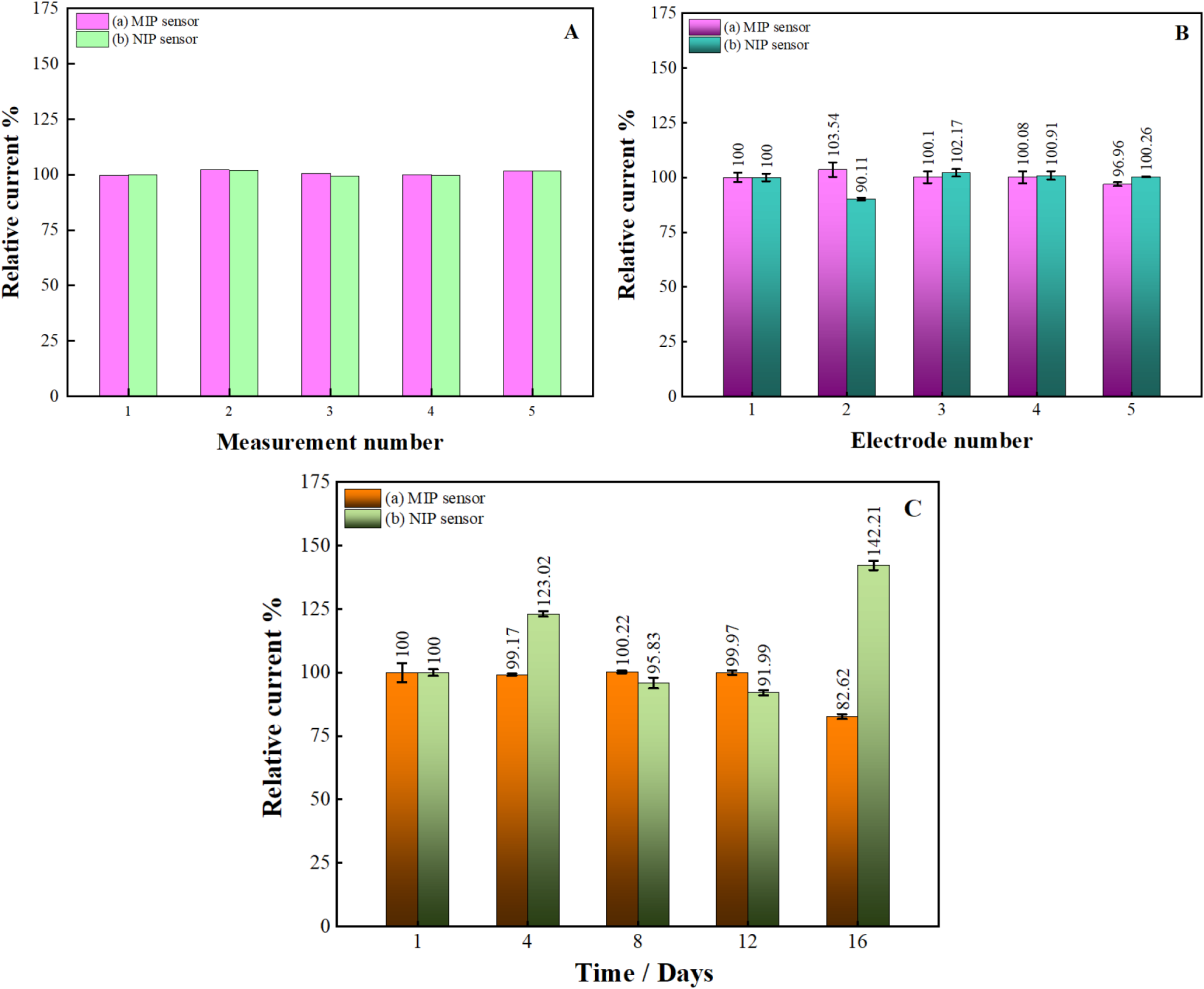
Bar graphs for (A) the reproducibility of the developed sensors for five different electrodes, (B) the reproducibility of the developed sensors for five consecutive measurements, and (C) the storage stability of the developed sensors for 16 days.

To investigate the compatibility of different electrodes with each other and the reproducibility of the sensor, five MIP and NIP sensors were prepared independently under optimum conditions, and DPV signals were recorded. The results are given in Figure 7B. It has been demonstrated that sensors with RSD values below 5% demonstrate excellent repeatability.^83^ The RSD values for the MIP and NIP sensors were calculated to be 2.33% and 4.93%, respectively. Although both sensors yielded a value of less than 5%, it can be stated that the MIP sensor demonstrated superior reproducibility.

Moreover, to determine the storage stability of the MIP and NIP sensors, the electrodes were stored at 4 °C following the initial measurement, and their sensitivity was analyzed by DPV measurements taken at four-day intervals. As illustrated in Figure 7C, the MIP sensor demonstrated remarkable stability against KYNA, retaining 82.62% of its initial activity after the 16th day. The MIP sensor was observed to remain functional for 8 days. The results obtained for the NIP sensor are not entirely consistent. As a consequence of this variability, the reliability of the results is compromised, and it can be concluded that the sensor lacks storage stability. It can thus be concluded that the MIP sensor displays superior storage stability in comparison to the NIP sensor. The specific cavities created in response to KYNA in the MIP sensor were also demonstrated to benefit electrode stability.

### 3.7 Real Monitoring of KYNA in Biological Fluids Using MIP and NIP Sensors

In order to assess the applicability of the developed MIP sensor in practical analyses, commercial serum samples were employed in real sample analysis, with measurements performed using the DPV method. The standard addition method was employed to ascertain the recovery values for the artificial fetal bovine serum and human serum media, as detailed in the section *real monitoring of KYNA in biological fluids using MIP and NIP sensors*. The resulting % recovery values are presented in Table 1. Upon analysis of the data presented in the table, it was found that the MIP sensor exhibited satisfactory recovery values in both experimental settings. This demonstrates that the MIP sensor can accurately determine KYNA in biological samples, thus indicating that the sensor has good practical applicability. The results demonstrate that the results obtained with the NIP sensor are not within acceptable limits. This demonstrates the efficacy of the sensor that has been developed.

**Table 1:** Real Sample Analysis Results in Biological Samples Using MIP and NIP Sensors

Samples	Sensors	Added amount (nM)	Found amount (nM)	Recovery (%)^a^	RSD (%)^a^
	MIP	100	100.387	100.39 ± 0.56	2.80
Fetal bovine serum	NIP	100	126.937	126.94 ± 0.64	9.09
Human serum	MIP	100	100.663	100.66 ± 0.57	2.83
	NIP	100	127.531	127.53 ± 0.31	6.83

a *N* = 3.

## 4 Conclusion

The necessity for the identification of a biomarker that can facilitate the early diagnosis of NDDs, such as AD, is becoming increasingly apparent. Among the potential biomarkers investigated in recent years, KYNA is among the most promising candidates. KYNA can serve as both a prognostic and a surrogate biomarker. MIP-based electrochemical sensors, which are relatively inexpensive, robust, and miniaturizable, have seen a notable increase in utilization, particularly in bioanalysis. Compared with conventional biorecognition systems, MIPs exhibit enhanced stability under harsh chemical or thermal conditions and are comparatively uncomplicated to synthesize. Nevertheless, they can result in heterogeneous binding sites that may affect binding affinity and reproducibility, and removal issues have the potential to compromise sensor performance. In this study, a series of optimization studies were conducted at each stage of the platform to eliminate the disadvantages. The present work presents an improved design of the selective, sensitive, low-cost KYNA analysis with an MIP-based sensor platform that increases sensitivity and selectivity in serum-based analysis. To the best of our knowledge, there is yet to be any existing literature on MIP-based KYNA biosensors. This study represents the first investigation of this subject. In contrast to previous studies, molecular imprinting was conducted using a one-step electropolymerization process, followed by removing the analyte with suitable solvents. According to the results, the linear working range for KYNA was 1 fM-500 nM, the LOD and LOQ were 0.278 fM and 0.928 fM, respectively. Developing a sensor capable of achieving an extremely low detection limit of 0.278 fM is attributed to the synergistic effect of two key elements: the high surface area and excellent conductivity of Cu–Ag bimetallic spheres, and the conducting polymer property of PEDOT. Furthermore, the MIP layer provides highly selective binding by forming KYNA-specific recognition cavities. Moreover, the KYNA MIP biosensor developed in this study was produced at a lower cost, with higher selectivity than those in the literature, and achieved without antibodies. The MIP sensor demonstrated operational stability for up to 8 days. The sensor exhibits excellent reproducibility and reusability. Concurrently, the sensor exhibits exceptional selectivity for KYNA in the presence of potential interfering molecules in biological fluids. In summary, the developed sensor facilitated the sensitive and precise determination of KYNA at femtomolar concentrations. Biological fluids substantiated the practical applicability of the sensor. It is further anticipated that the MIP-based methodology developed in this project can be employed for the sensitive and selective determination of other biomarkers at femtomolar levels, which can potentially be utilized for the early diagnosis of AD. The sensor’s simple platform and manufacturing process, coupled with its high analytical sensitivity, mean that it is well suited for development into a portable diagnostic system for the early detection of Alzheimer’s disease. However, further technical improvements are required before a system can be successfully commercialized. These include automated sample preparation, a portable electrochemical reader, a user-friendly software interface, and a wireless data transfer infrastructure. The sensor will be marketed as a kit after the successful completion of clinical validation studies, stability testing, and regulatory compliance. The commercialization of the sensor is planned to complete these processes in the future.

## Supplementary Material



## Data Availability

The data supporting this study’s findings are available in the Supporting Information of this article.

## Notes

The tables present the electrochemical characteristics and electroactive surface area of bare and modified electrodes; they also compare analytical parameters in different sensors; the plot of peak current versus the square root of scan rate for all electrodes was presented, along with graphs drawn to optimize experimental conditions (PDF)
