# Supplementary material for: Fabrication of MIP-based sensors for femtomolar detection of kynurenic acid for early diagnosis of neurodegenerative diseases
Source: ACS Omega. 2025 May 16;10(20):20907–21. doi: 10.1021/acsomega.5c02339 (PMC12120629; doi:10.1021/acsomega.5c02339)
Supplement: Supplementary file 1 [file ao5c02339_si_001.pdf]

Supplementary Material

Fabrication of MIP-based sensor for femtomolar detection of kynurenic acid for early diagnosis of neurodegenerative diseases

Kübra TURAN[1], Gözde AYDOĞDU TIĞ[1]*
[1] Ankara University, Faculty of Science, Department of Chemistry, Ankara, 06100, Türkiye

Table S1. Electrochemical characteristics for bare and modified electrodes.

Electrode	E_{pa} / mV	E_{pc} / mV	i_{pa} / µA	i_{pc}/ µA	ΔE_p / mV	Δi_p / µA
**Bare GCE**	351.87	132.14	44.51	-48.16	219.73	92.67
**Cu/GCE**	342.10	266.42	80.14	-93.41	75.68	173.55
**Cu-Ag BS/GCE**	293.27	210.27	93.91	-101.35	83.00	195.26
**PEDOT-KYNA/Cu-Ag BS/GCE**	284.12	130.31	4.98	-15.74	153.81	20.72
**MIP/Cu-Ag BS/GCE**	310.36	173.65	74.62	-80.86	136.71	155.48
**KYNA/MIP/Cu-Ag BS/GCE**	306.49	154.90	60.33	-69.04	151.59	129.37
**NIP/Cu-Ag BS/GCE**	319.30	164.49	60.30	-65.01	154.81	125.31

Table S2. Electroactive surface area for bare and modified electrodes.

Electrodes	Electroactive surface area (cm^2)
Bare GCE	0.0714
Cu/GCE	0.0824
Cu-Ag BS/GCE	0.113
PEDOT-KYNA/Cu-Ag BS/GCE	0.0203
MIP/Cu-Ag BS/GCE	0.104
KYNA/MIP/Cu-Ag BS/GCE	0.0873
PEDOT/Cu-Ag BS/GCE	0.0906
NIP/Cu-Ag BS/GCE	0.0924
KYNA/NIP/Cu-Ag BS/GCE	0.0978

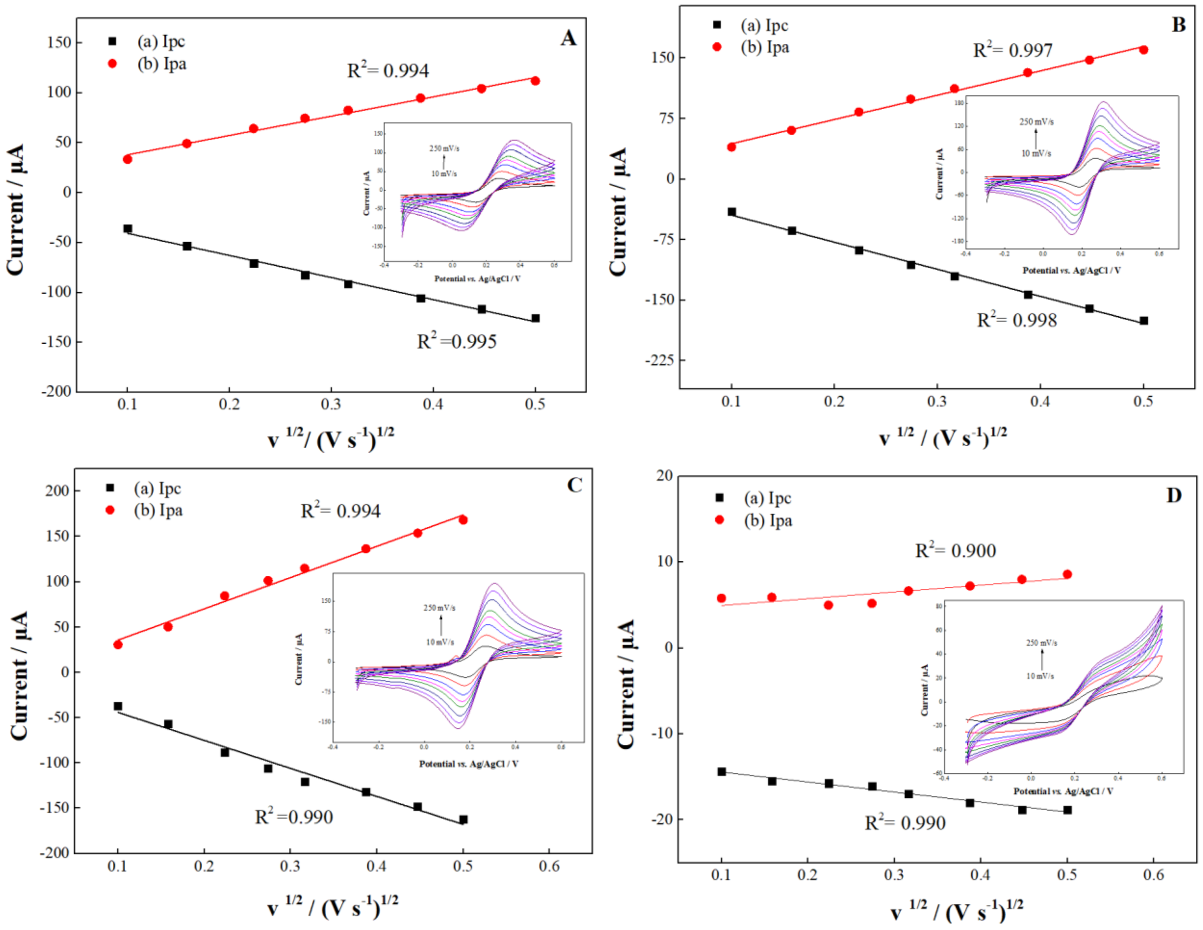

A
B
C
D
(a) Ipc
(b) Ipa
R2= 0.994
R2 =0.995
R2= 0.997
R2= 0.998
R2= 0.994
R2 =0.990
R2= 0.900
R2= 0.990
Current / μA
v 1/2/ (V s-1)1/2
250 mV/s
10 mV/s
Potential vs. Ag/AgCl / V

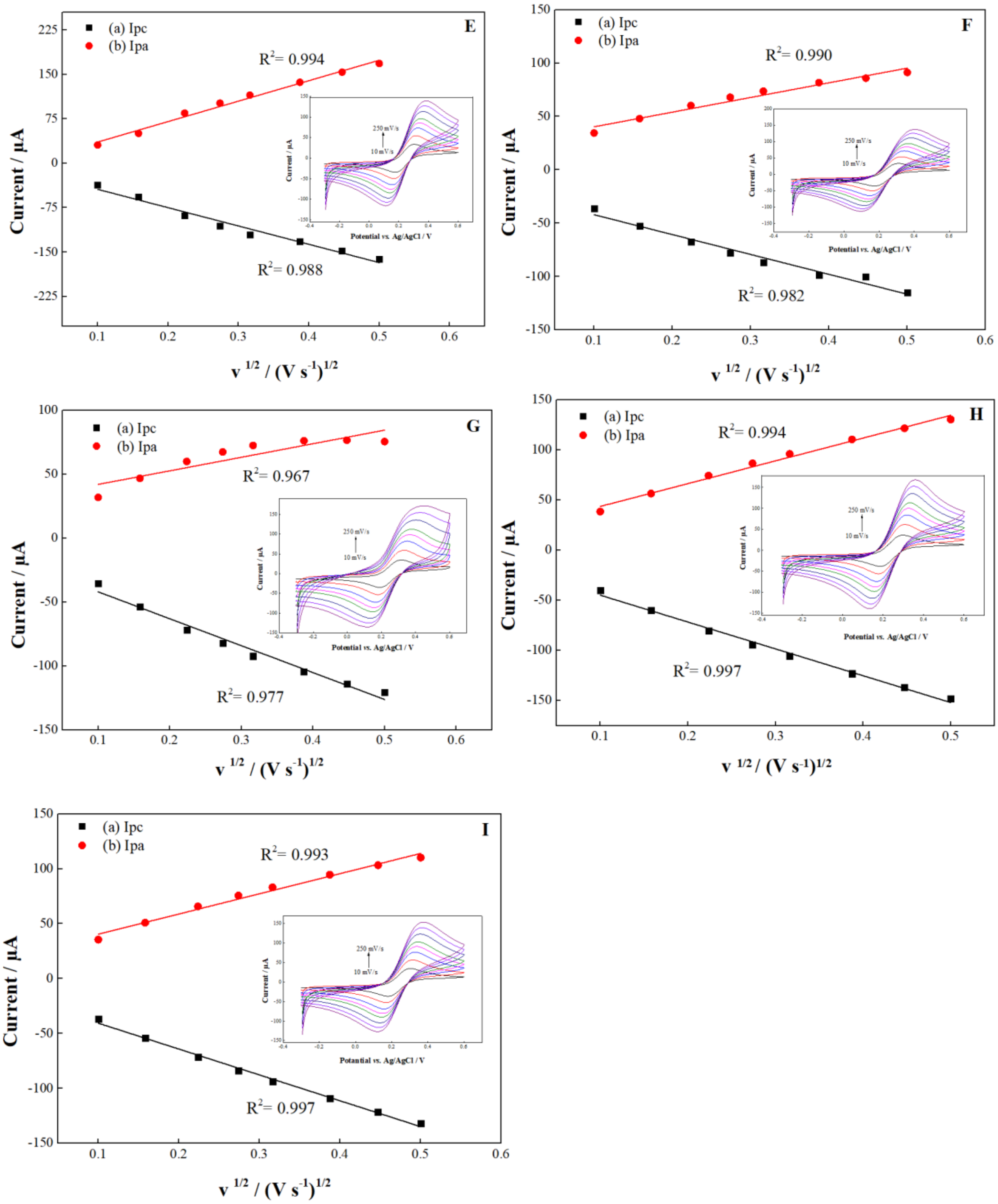

Figure S1. The plot of the peak current *vs.* the square root of the scan rate for (A) Bare GCE, (B) Cu/GCE, (C) Cu-Ag BS/GCE, (D) PEDOT/KYNA/Cu-Ag BS/GCE, (E) MIP/Cu-Ag BS/GCE, (F) KYNA/MIP/Cu-Ag BS/GCE, (G) PEDOT/Cu-Ag BS/GCE, (H) NIP/Cu-Ag BS/GCE ve (I) KYNA/NIP/Cu-Ag BS/GCE (inset: CVs at different scan rates).

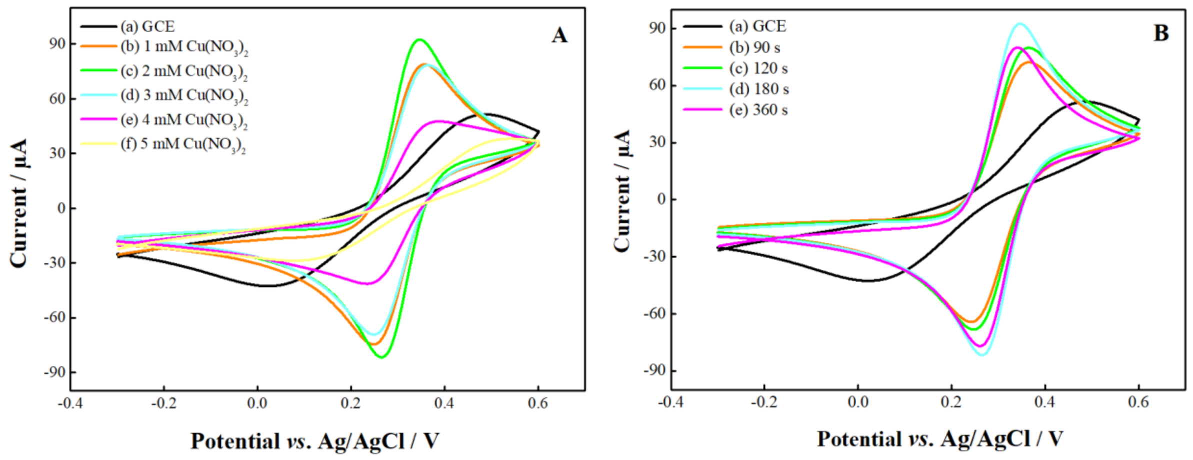

Figure S2. (A) Cyclic voltammograms of Cu/GCE electrodes prepared different concentrations at (a) Bare GCE ve (b) 1 mM $Cu(NO_3)_2$, (c) 2 mM $Cu(NO_3)_2$, (d) 3 mM $Cu(NO_3)_2$, (e) 4 mM $Cu(NO_3)_2$, ve (f) 5 mM $Cu(NO_3)_2$ (scan rate 50 mV/s, in 0.1 M KCl solution containing 5.0 mM $Fe(CN)_6^{3-/4-}$) and **(B)** Cyclic voltammograms of Cu/GCE electrodes prepared different electrodeposition time at (a) bare GCE ve (b) 90, (c) 120, (d) 180, and (e) 360 seconds (scan rate 50 mV/s, in 0.1 M KCl solution containing 5.0 mM $Fe(CN)_6^{3-/4-}$).

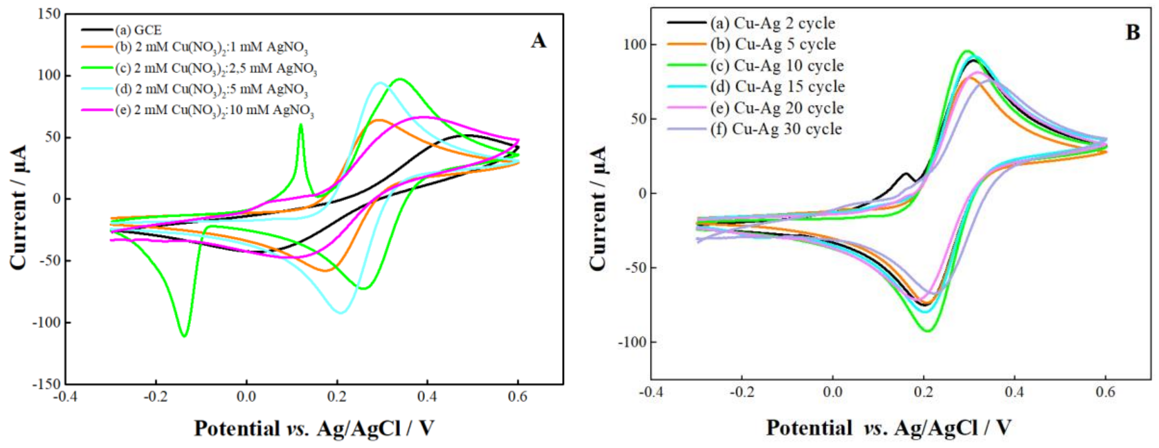

Figure S3. (A) Cyclic voltammograms of Cu-Ag BS/GCE electrodes prepared different concentrations at (a) bare GCE ve (b) 2 mM $Cu(NO_3)_2$:1 mM $AgNO_3$, (c) 2 mM $Cu(NO_3)_2$:2,5 mM $AgNO_3$, (d) 2 mM $Cu(NO_3)_2$:5 mM $AgNO_3$, ve (e) 2 mM $Cu(NO_3)_2$:10 mM $AgNO_3$ (scan rate 50 mV/s, in 0.1 M KCl solution containing 5.0 mM $Fe(CN)_6^{3-/4-}$) and **(B)** Cyclic voltammograms of Cu-Ag BS/GCE electrodes prepared at (a) 2 ve (b) 5, (c) 10, (d) 15, (e) 20 and (e) 30 cycles (scan rate 50 mV/s, in 0.1 M KCl solution containing 5.0 mM $Fe(CN)_6^{3-/4-}$).

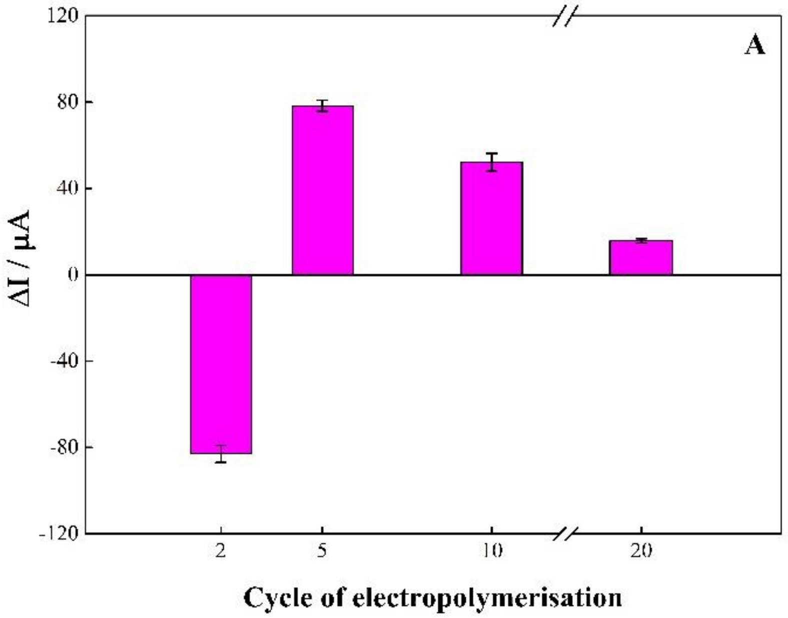

A
ΔI / µA
120
80
40
0
-40
-80
-120
2
5
10
20
Cycle of electropolymerisation

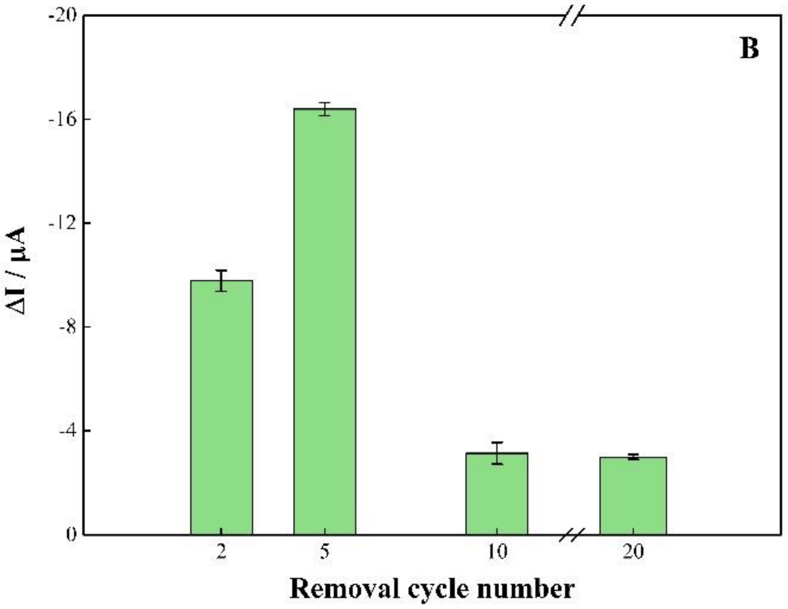

B
ΔI / µA
-20
-16
-12
-8
-4
0
2
5
10
20
Removal cycle number

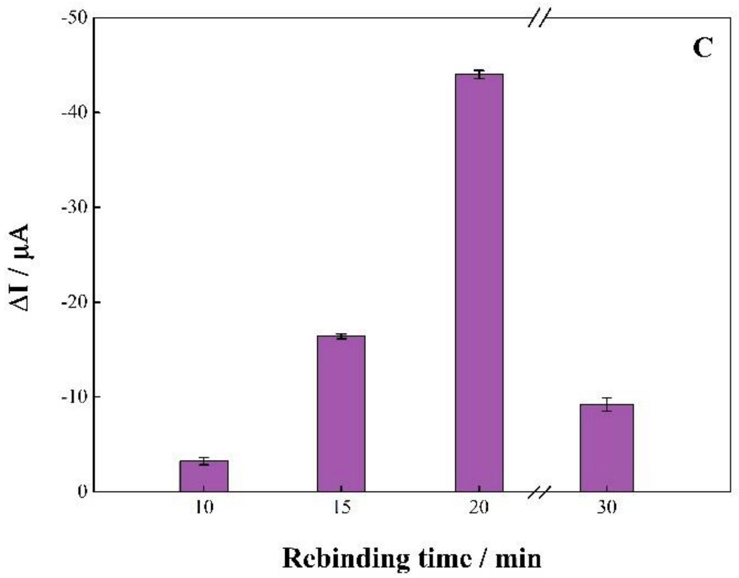

Figure S4. (A) Effect of different numbers of electropolymerization cycles on the current response. **(B)** Optimization of the number of cycles in the KYNA removal step. **(C)** Optimization of the KYNA rebinding time after the removal step.

Table S3. A comparative of analytical parameters in various sensors for determining KYNA and other biomarkers of neurological diseases.

**Biomarkers**	**Sensor platform**	**Method**	**Linear range**	**LOD**	**Ref.**
**mirR-137**	SPCE/ERGO+AuNWs	DPV	5-750 fM	1.7 fM	[5]
**AβO**	AgNPs/HCR/Apt2/AβO/ MCH/Apt1/GE	LSV	1 pM-10 nM	430 fM	[69]
**Aβ**	Au-FLGN Elektrot/Aptamer/MCH/Redox marker/ Aβ	DPV	0.002–1.28 ng/mL	0.4 pM	[72]
**Aβ**	HRP-DAb-tau-CAb-3D-Au-PAMAM-ρ-AB	CV, EIS	10-100 pg/mL	1.7 pM	[73]
**p53**	SPCE/Antibody/BSA/p53-biotin/S-AP	LSV	2-50 nM	0.05 nM	[74]
**KYNA**	GE/MU/MUA/BSA-pseudo-KYNA/Secondary-Ab/HRP	CV/CA/EIS	10 pM-100 nM	16.9 pM	[9]
**KYNA**	MIL-101(Cr)-CPE	DPV	100-150000 nM	17 nM	[71]
**CDNF protein**	CDNF/MIP	VIS-ellipsometry	5-50 ng/mL	4.2 nM	[75]
**β-amyloid-42**	NCd/PANI/Au-SPE MCd/PANI/Au-SPE	SWV/EIS	-	0.44 pM	[76]
**β-amyloid42**	MPan/CNT-Cu/C-SPE	SWV	1-66 nM	0.400 pM	[77]
**β-amyloid42**	MIP/Aβ42/PEDOT/CI-HME	SWV	0.1 nM-1 µM	0.0670 nM	[78]
**β-amyloid42**	MIP/d-$Ti_3C_2T_X$/MWCNTs/GCE	EIS/CV	1-100 fM	0.3 fM	[70]
**miRNA-34a**	GO/PGE	EIS	0-10 µM	1.9 µM	[79]
**miRNA-34a**	CA/GO/PGE	DPV	5-35 µM	7.52 µM	[80]
**KYNA**	MIP/Cu-Ag BS/GCE	DPV	1 fM-500 nM	0.278 fM	**This study**

AgNPs: Silver Nanoparticles, AβO: Amyloid β-peptide oligomer, Apt 1: Aptamer 1, Apt 2: Aptamer 2, AuNWs: Gold Nanowires, BSA: Bovine Serum Albumin, CAB: Capture Antibody, CDNF: cerebral dopamine neurotrophic factor, DAb: Labeled Detector Antibody, DPV: Differential Pulse Voltammetry, ERGO: Reduced Graphene Oxide, GA: Glutaraldehyde, GCE: Glassy Carbon Electrode, GE: Glassy Electrode, GO: Graphene Oxide, HCR: Hybridization Chain Reaction, HRP: Horseradish Peroxidase, LSV: Linear scanning voltammetry, MCH: Mercapto-l-hexanol, miRNA-34a: MicroRNA-34a, MU: 11-Mercapto-1-undecanol, MUA: 11-Mercaptoundecanoic acid, MWCNTs: Multi-Walled Carbon Nanotubes, PAMAM: Polyamidoamine, PEDOT: Poly(3,4-Ethylenedioxythiophene), PGE: Pencil Graphite Electrode, S-AP: Streptomyces avidin labeled with alkaline phosphatase, SPCE: Screen Printed Carbon Electrode, SPE: Screen Printed Electrode, SWV: Square Wave Voltammetry
